# Comparative genomic analysis of fungal genomes reveals intron-rich ancestors

**DOI:** 10.1186/gb-2007-8-10-r223

**Published:** 2007-10-19

**Authors:** Jason E Stajich, Fred S Dietrich, Scott W Roy

**Affiliations:** 1 Department of Molecular Genetics and Microbiology, Center for Genome Technology, Institute for Genome Science and Policy, Duke University, Durham, NC 27710, USA; 2 Miller Institute for Basic Research and Department of Plant and Microbial Biology, 111 Koshland Hall #3102, University of California, Berkeley, CA 94720-3102, USA; 3 National Center for Biotechnology Information, National Library of Medicine, National Institutes of Health, 8600 Rockville Pike, Bethesda, MD 20894, USA

## Abstract

**Background:**

Eukaryotic protein-coding genes are interrupted by spliceosomal introns, which are removed from transcripts before protein translation. Many facets of spliceosomal intron evolution, including age, mechanisms of origins, the role of natural selection, and the causes of the vast differences in intron number between eukaryotic species, remain debated. Genome sequencing and comparative analysis has made possible whole genome analysis of intron evolution to address these questions.

**Results:**

We analyzed intron positions in 1,161 sets of orthologous genes across 25 eukaryotic species. We find strong support for an intron-rich fungus-animal ancestor, with more than four introns per kilobase, comparable to the highest known modern intron densities. Indeed, the fungus-animal ancestor is estimated to have had more introns than any of the extant fungi in this study. Thus, subsequent fungal evolution has been characterized by widespread and recurrent intron loss occurring in all fungal clades. These results reconcile three previously proposed methods for estimation of ancestral intron number, which previously gave very different estimates of ancestral intron number for eight eukaryotic species, as well as a fourth more recent method. We do not find a clear inverse correspondence between rates of intron loss and gain, contrary to the predictions of selection-based proposals for interspecific differences in intron number.

**Conclusion:**

Our results underscore the high intron density of eukaryotic ancestors and the widespread importance of intron loss through eukaryotic evolution.

## Abstract 2

Analysis of intron gain and loss in fungal genomes provides support for an intron-rich fungus-animal ancestor.

## Background

Unlike bacteria, the protein-coding genes of eukaryotes are typically interrupted by spliceosomal introns, which are removed from gene transcripts before translation into proteins. Eukaryotic species vary dramatically in their number of introns, ranging from a few introns per genome to several introns per gene. The reasons for these vast differences, as well as the explanation for the particular pattern of intron number across species, remain obscure. The first genomes with characterized intron densities suggested the possibility of a close association between intron number and organismal complexity. The initial animal and land plant species studied had high intron densities, for instance, *Homo sapiens *with 8.1 introns per gene [1], *Caenorhabditis elegans *with 4.7 [2], *Drosophila melanogaster *with 3.4 [3], and *Arabidopsis thaliana *with 4.4 [4]. By contrast, many unicellular species were found to have few [5]. However, further studies have shown high intron densities in a variety of single-celled species [6,7], with great variation in intron density within eukaryotic kingdoms.

The case of fungi is particularly striking. The first fungal genomes characterized, the yeasts *Schizosaccharomyces pombe *(0.9 per gene) [8] and *Saccharomyces cerevisiae *(0.05 per gene) [9], have low intron densities. However, the euascomycete fungi *Neurospora crassa *and *Aspergillus nidulans *have much higher intron densities (2-3 per gene) [10,11], and intron densities in basidiomycete and zygomycete fungi are among the highest known among eukaryotes (4-6 per gene) [12,13]. Gene structures among fungal species are known to differ between closely related *Cryptococcus *species [14] or more distantly related euascomycete species [15]. Conservation of intron positions between deeply diverged fungal groups has not been systematically evaluated, and it is not known whether the large numbers of introns among these major fungal lineages are due primarily to retention of introns present in fungal ancestors or to intron gain into ancestrally intron-poor genes.

Many intron positions are shared between eukaryotic kingdoms. In particular, many intron positions are shared between plants and animals but not the intron-sparse fungi *S. pombe *and *S. cerevisiae*, a pattern that is due to some combination of loss in fungi [16-19], and homoplastic insertion in plants and animals [16,17]. Separate analyses have supported different pictures, either of moderate ancestral intron densities followed by a tripling of intron number in vertebrates and plants [16,17,19], or of high ancestral intron density and massive intron loss in *S. pombe*, *S. cerevisiae*, and a variety of other species [18,20]. This study represents the first multi-kingdom comparative analysis to include multiple diverse and intron rich fungi, permitting a more accurate reconstruction of intron evolution through fungal history.

We used comparative genomic analysis of the gene structures of 1,161 sets of orthologs among 21 fungal species and four outgroups. We found that studied fungal species share many intron positions with distantly related species; both the fungal ancestor and fungus-animal ancestor (Opisthokont) were very intron rich, with intron densities matching or exceeding the highest known average densities in modern species of fungi and approaching the highest known across eukaryotes. Fungal evolution has been dominated by intron loss and we identify independent nearly complete intron loss along three distinct fungal lineages in addition to overall patterns of intron loss.

## Results and discussion

### Intron position data set

**Figure 1:**
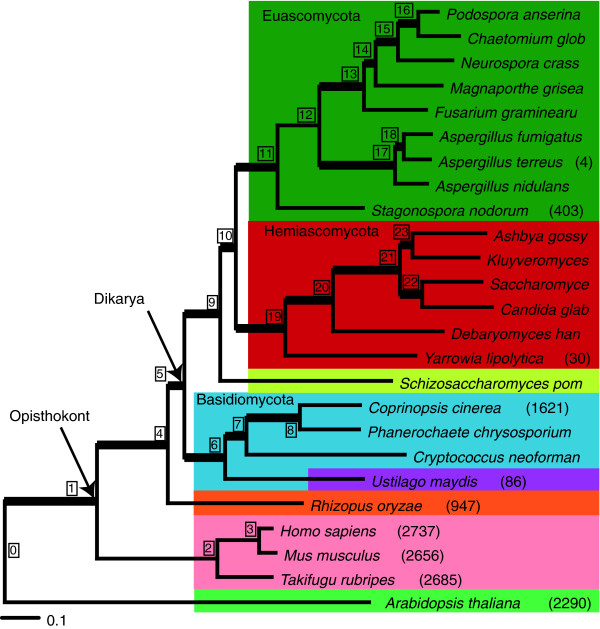
This figure depicts a phylogenetic tree of the species used for this analysis. The tree is based on Bayesian phylogenetic reconstruction of 30 aligned orthologous proteins from the 25 species. The numbers after the species names list the total number of introns present in the CORs for each species. *U. maydis *is colored purple to indicate it has a different intron pattern than the rest of the basidiomycete fungi sampled. Numbers in boxes are node numbers that are used in Tables seen Additional data files 4 and 5.

**Figure 2:**
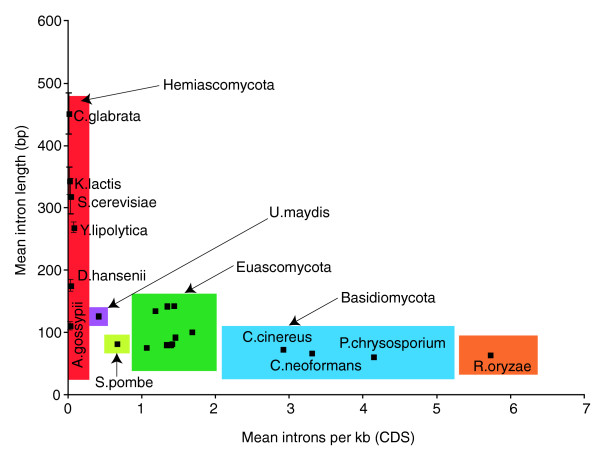
Intron length versus average number of introns per kilobase. Colored boxes indicate the fungal clade as shown in Figure 1: red, Hemiascomycota; yellow, Archiascomycota; green, Euascomycota; orange, Zygomycota; blue, Basidiomycota; purple, basidiomycete *U. maydis*. Bars indicating standard deviation in intron length are drawn but only visible for the intron-poor species. CDS, coding sequence.

To study fungal intron evolution, we identified 1,161 orthologs among 21 fungal species and 4 outgroups (Figure 1; see Materials and methods). We aligned the amino acid sequences and mapped the corresponding intron positions onto the alignments. There were a total of 7,535 intron positions in 4.15 Megabases of conserved regions of alignment (hereafter 'conserved orthologous regions' (CORs)). Species' intron counts ranged from 0.001 introns per kilobase (kb) in CORs (in *S. cerevisiae *with 7 total introns) to 6.7 introns per kb (2,737 introns in humans; Figure 1). Figure 2 summarizes the average number of introns per kb of coding sequence versus median intron length. In general, major lineages are clearly separated by intron density. One exception is *Ustilago maydis*, a basidiomycete fungus that has many fewer introns than other members of its clade. Median intron length is inversely and significantly correlated with the average number of introns per kb (R^2 ^= 0.23, *P *= 1e^-4^; Spearman correlation coefficient), although the trend is not significant when the hemiascomycete fungi are excluded (R^2 ^= 0.18, *P *= 0.06). This finding of much longer introns in the very intron-poor hemiascomycetes is intriguing, particularly in light of other peculiarities of evolution in very intron poor lineages [21]. In particular, very intron-poor lineages, including hemiascomycetes (see below), have more regular 5' intronic sequences (that is, a stronger consensus sequence at the beginning of introns). Presumably, this conservation of 5' boundaries facilitates intron splicing, in which case increased intron length might be better accommodated. Comparison between other very intron-poor species and more intron-rich relatives should yield insight into the peculiarities of evolution of very intron-poor lineages. Additional data file 4 provides the summary statistics of coding sequence, intron length, and density for the sampled fungal genomes.

### Patterns of intron sharing

**Figure 3:**
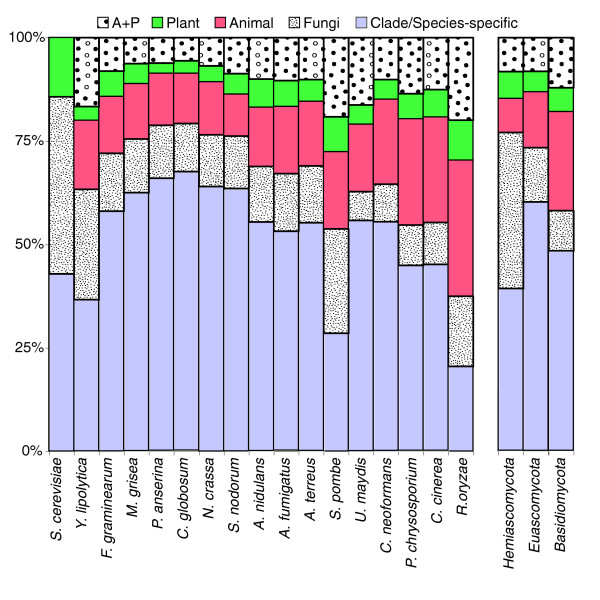
Pattern of intron sharing of fungal species. Fractions of intron positions that are shared with animal or plant (A+P), plant, animal, with another fungal clade (Euascomycota, Hemiascomycota, or Basidiomycota), or specific to the species or clade.

**Figure 4:**
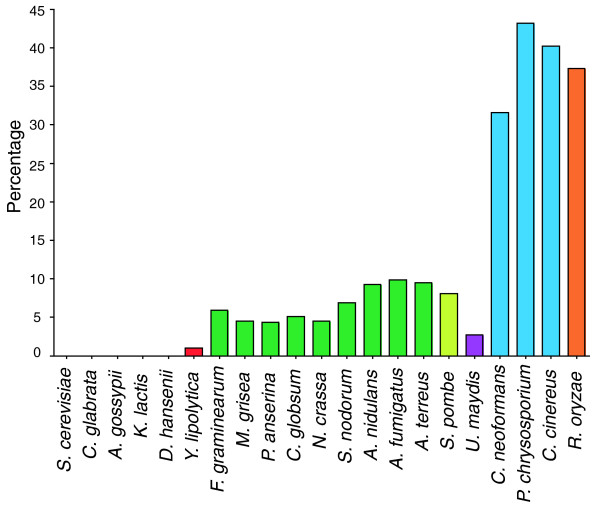
Fraction of shared plant-animal intron positions in each fungal species. Among the 501 intron positions that are shared between *A. thaliana *and a vertebrate (and thus likely present in the fungus-animal ancestor), the fraction that is shared with each fungal species is given. Color coding is lavender: introns found only within the clade or a single species, maroon: introns shared only with other fungi,, pink: introns shared with animals, green: introns shared with plants (*A. thaliana*), brown: introns shared with animals or plants.

Patterns of intron position sharing vary across fungal species. Excluding the extremely intron-poor Hemiascomycota clade, species show between 3.7% and 38.7% species-specific intron positions, while between 32.0% and 76.5% of introns are shared with a species outside of the clade (different colors in Figure 1), and between 20.5% and 60.1% are shared with a non-fungal species. Figure 3 summarizes the pattern of species-specific and shared intron positions across the CORs. Out of 7,535 intron positions, 3,307 are species-specific positions, 1,602 of which are specific to *A. thaliana*. Of the 501 intron positions shared between plants and animals, from 2.76% in *U. maydis *to 43.2% in *Phanerochaete chrysosporium *(Figure 4) are shared with the various fungal species. In all, 60.7% of shared plant-animal positions are also represented in at least one fungal species.

Species within a clade share more intron positions than between clades. Another way to visualize this is using a phylogenetic tree derived from a parsimony analysis where each intron position is a binary character (Additional data file 1). We constructed a phylogenetic tree using Dollo parsimony [22,23] from the intron presence absence matrix for the CORs. Dollo parsimony assumes that 0 to 1 transitions (intron gain) can occur only once across the tree for each site, and then infers a minimum number of 1 to 0 transitions (intron loss) to explain each phylogenetic pattern. Surprisingly, our species tree and parsimony tree from the intron position matrix provide nearly the same result, with two exceptions: the unresolved hemiascomycetes, which have few intron presence characters; and the position of *U. maydis *and *S. pombe*, presumably due to a high degree of intron loss in those lineages. Previous failed attempts to reconstruct phylogeny by applying parsimony analysis to intron positions experienced a similar phenomenon, with intron poor taxa artificially grouping together [19]. As such, it seems possible that intron positions could be good phylogenetic characters in slowly evolving taxa, but will likely encounter problems in cases of widespread intron loss.

### High ancestral intron number and ongoing loss and gain

**Figure 5:**
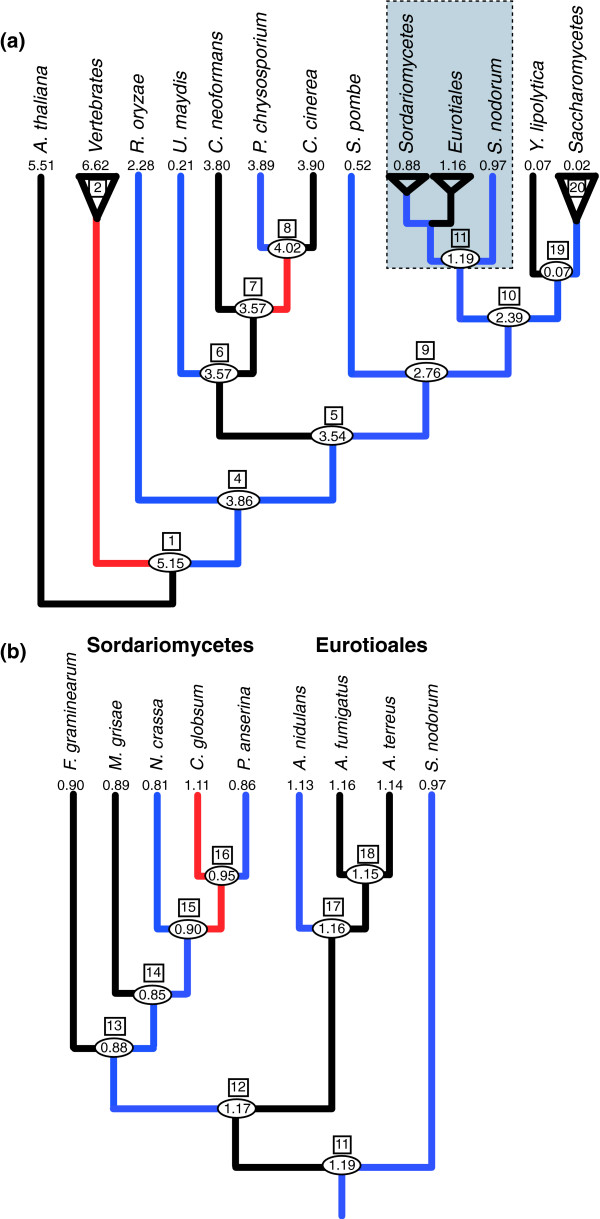
Estimated number of introns per kilobase in CORs through fungal history using the EREM method. Numbers in ovals give estimated ancestral values normalized by the total number of aligned bases in the CORs (4.15 Mb). Numbers in black boxes represent the node number references in the tables in Additional data files 4 and 5. Blue branches indicate two or more estimated losses for each estimated gain; red > 1.5 gains per loss. **(a) **Summarized fungal tree. Triangles indicate clades, with values for the clade ancestor indicated. **(b) **Introns per kilobase through Euascomycota history, the clade indicated by the grey box in (a).

We next studied intron loss and gain in fungi in CORs of 1,161 genes. Four previously proposed methods showed very similar pictures, with large numbers of introns present in ancestral genomes and widespread subsequent intron number reduction along various fungal lineages (Figure 5, and tables in additional files 4 and 5). We find that the fungal ancestor was at least as intron rich as any modern fungal species and that the fungus-animal ancestor was 25% more intron-rich than any modern fungus, with at least three-quarters as many introns as modern vertebrates.

Intron number reduction has been a general feature of fungal evolution (Figure 5). We estimate that at least half of the studied fungal lineages (excluding hemiascomycetes) experienced at least 50% more losses than gains, while only between three and six experienced 50% more gains than losses (Figure 5; depending on method used, see Additional file 5). Dramatic intron reduction has occurred within each fungal clade. *U. maydis' *0.21 introns per kb represent a 94% reduction in intron number relative to the basidiomycete ancestor; since the ascomycete ancestor (with at least 2.77 introns per kb), hemiascomycetes (0.01-0.07 introns per kb) species have reduced their intron number by at least 94%, *S. pombe *has reduced its intron number by 81% (0.52 introns per kb), and even relatively intron-rich euascomycete species (0.81-1.16 introns per kb) have undergone a 60% reduction in intron number. Interestingly, following dramatic intron number reduction in the euascomycete ancestor, intron number has remained relatively unchanged within the clade (Figure 5b), consistent with previous results [15,24].

On the other hand, our results also attest to ongoing intron gain. Most species have experienced hundreds of intron gains in CORs (although many have subsequently been lost) since the fungal ancestor, and nearly every studied species is estimated to have gained more than one intron per kb since the intron ancestor. Differences in intron gain are sometimes the central determinant of modern differences in intron number. For instance, *S. pombe *shares as many of the 507 intron positions shared between plants and animals (most of which are likely ancestral) as most euascomycetes; euascomycete species' 50-100% more introns than *S. pombe *are thus primarily due not to greater retention of ancestral introns but to recent gain. Likewise, *Cryptococcus neoformans *retains fewer shared plant-animal introns than does *Rhizopus oryzae*, yet has 70% more introns, apparently due to more intron gain.

### Intron evolution in hemiascomycetes

Intron evolution within hemiascomycetes provides insights into the evolution of nearly intronless lineages. The extensive loss of introns in hemiascomycetes corresponds to the position in the fungal phylogeny with a significant shift in intron structure. Intron structure in hemiascomycetes requires a six base sequence at the 5' splice site and a seven base pair site at the branching point [25]. The other sampled fungi require only a limited intron splice consensus at the 5' splice site and branching point. Previous results have shown that this correspondence between greatly reduced intron number and stronger conservation of intron boundaries across eukaryotes is a general trend [21]. Two explanations have been proposed. Irimia *et al*. [21] suggested that mutations that led to stricter sequence requirements by the spliceosome might be favored in intron-poor but not intron-rich species, in which case widespread intron loss would lead to increased strictness of splicing requirements (and thus intron boundaries). Another possibility [26] is that a shift in splicing mechanism, requiring more extensive conserved sequences at the branch point and 5' splice junction, would create a condition where introns would be more deleterious due to the additional sequence constraint necessary for splicing. In this case, increased strictness of splicing requirements (and thus intron boundaries) would drive intron loss.

Why have all of the introns then not been lost in hemiascomycete species? Some of the *S. cerevisiae *introns encode functional elements such as small nucleolar RNAs (snRNAs) [27] or promoter elements [28]. snRNAs located in the introns of ribosomal proteins are found in orthologous loci of basidiomycetes and ascomycetes (for example, snR39 in RPL7A of *S. cerevisiae*), indicating their conservation since divergence from the fungal ancestor. However, only 8 of 76 snRNAs are found in the 275 nuclear introns in *S. cerevisiae *[9]. Introns also play a role in regulation of RNA and proteins [29], perhaps through a role in recruiting factors that mediate splicing-dependent export [30]. Some of the remaining introns in hemiascomycetes may also provide a necessary role as *cis*-regulatory containing elements or encoding factors necessary for post-transcriptional regulation, but they may also persist by chance due to low rates of loss.

On the other hand, our results show that hemiascomycete intron positions are not in general widely shared. Only one of the seven intron positions in non-*Yarrowia lipolytica *hemiascomycete species examined is shared with any species more distant than euascomycetes. However, six of the seven are broadly shared within the hemiascomycete lineage, suggesting either that the remaining introns are very hard to lose or that loss rates have greatly diminished within the lineage. By contrast, 14 of 23 introns present in *Y. lipolytica *but no other hemiascomycete are shared with a non-euascomycete, and 10 are shared with plants and/or animals; thus, widely shared introns have been preferentially lost among hemiascomycetes after the divergence with the *Y. lipolytica *ancestor.

### Selection and intron evolution

Eukaryotic species vary in their numbers of introns by orders of magnitude. These differences have traditionally been attributed to alleged differences in the intensity of selection against introns across eukaryotes [31,32]. Additionally, it has been proposed that selection against introns could be similar, with differences in population size determining intron number [33,34]. Under these models, lineages with strong selection against introns (or large population size) should experience low rates of intron gain and high rates of intron loss. Lineages with weaker selection (or smaller population size) should experience more intron gain and less intron loss. Both models thus predict a strong inverse correlation between intron gain and loss rates. However, the data presented here show no clear pattern of inverse correlation (Figure 5).

### On the reconstruction of intron evolution

These results provide an excellent opportunity to compare different previously proposed methods for reconstruction of intron evolution. There are five previously proposed methods. Dollo parsimony assumes a minimal number of changes but that once an intron is lost at a position, it is never regained [22]. Roy and Gilbert's method ('RG') [18,20] assumes that all intron positions shared between species are representative of retained ancestral introns, while the methods of Csűrös [16] and of Nguyen and coauthors ('NYK') [17] allow multiple intron insertions into the same site, so-called 'parallel insertion'. Carmel and coauthors' [35] method additionally allows for the possibility of heterogeneity of rates of both intron loss and gain across sites.

Previously, application of four methods (Dollo, RG, Csűrös, and NYK) to intron positions in conserved regions of 684 sets of orthologs showed very different pictures of early eukaryotic evolution. Roy and Gilbert estimated the animal-fungus and plant-animal ancestors had some three-fifths as many introns as vertebrates (among the most intron-dense known modern species) [18], while Rogozin and collaborators [19], Csűrös [16], and Nguyen and collaborators [17] all concluded that these ancestors had only half that many introns, and that higher intron densities in plants and vertebrates were due to dramatic increases in intron number. This difference has repeatedly been attributed to overestimation by the RG method [16,17,36,37], and the RG estimates have been called 'drastic' and 'generous' [27,28]. The rationale for this conclusion has been that if a significant number of matching intron positions represent parallel insertion, the RG method will clearly overestimate ancestral intron number.

We used all five methods to reconstruct intron evolution for the current data set. In contrast to the previous discordance, all methods now provide similar estimates for the numbers of introns in the animal-fungus ancestor. Dollo parsimony tended to be very different from the rest of the estimates for deep nodes in the tree. The Carmel and NYK methods show the most striking agreement, with less than 2% difference across all nodes except for the Opisthokont ancestor (3.3% difference). The NYK and Csűrös methods also show striking agreement, giving estimates within 2% of each other for 13 out of 18 (non-hemiascomycetes) nodes, and to within 10% for 17 out of 18. The RG method agreed with the other three methods to within 15% for all nodes except six and was not more than 30% higher than either of the other methods for any node other than the Ascomycete node. Notably, the three nodes on which RG was comparatively highest for the current data set are deep nodes near very long branches in this tree. Thus, further taxonomic sampling would likely bring even these nodes into better agreement (see below). Numbers of intron losses and gains in CORs along each branch were also estimated using all four methods. Though absolute numbers of estimated intron losses and gains along each branch varied more considerably between methods, there was a striking agreement in the relative incidence of intron loss and gain, with Csűrös (2.03 losses per gain), evolutionary reconstruction by expectation-maximization (EREM; 2.14) and NYK (2.12) nearly identical and RG only 21% higher (2.66). Notably, overall estimated numbers of gains were very similar, with only 19 more gains by RG than NYK. Results for all methods are given in Additional data files 4 and 5.

Strikingly, all four methods now estimate that the fungus-animal ancestor had at least 70% as many introns as vertebrates, 15% more than estimated by Roy and Gilbert and more than twice that previously estimated by Csűrös and NYK. Thus, it appears that the previous difference in estimated intron density in the animal-fungal ancestor was not due to overestimation by the RG method, but to a 2.5-fold underestimation by the other methods. Indeed, even the estimates of Roy and Gilbert appear to have been conservative [20].

Why should this be? Following the original authors [20], we suggest that this pattern may be due to unrecognized differences in rates of intron loss across sites. Clear differences in rates of intron loss across sites (that is, different rates of loss for introns at different positions along the same lineage) have been observed over both short [38,39] and long [40,41] evolutionary timescales; however, three out of four methods fail to take into account such differences in loss rate. Given the recurrent finding of differences in intron loss rates in a variety of studies, it is interesting that Carmel and coauthors' recent work did not find significant differences in rates, and that their method so closely cleaves to the findings of the other methods described here. Clearly, more study into possible differences in rates of evolution across sites, and their effects on current methods, is necessary.

**Figure 6:**
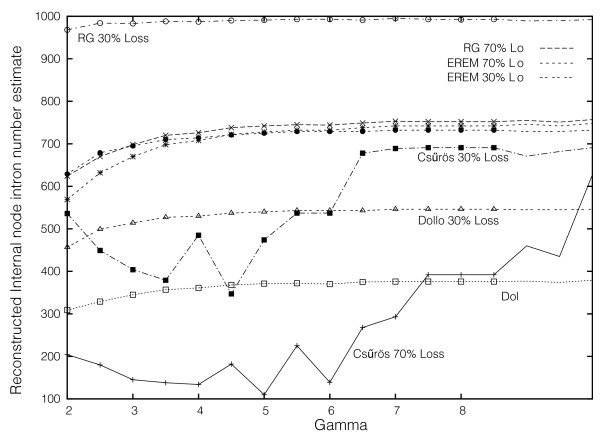
Performance of Csűrös, RG, Dollo parsimony, and EREM methods for the four-taxa case under intron loss rate variation with loss rates given by a standard gamma distribution with indicated alpha value, in which 30% or 70% of introns are lost along each external branch. The actual number of simulated ancestral intron numbers is 1,000; thus, both Csűrös and Dollo methods underestimate ancestral density under all cases. The relevant phylogeny is given in Additional file 2.

We performed simulations of intron evolution that included variations in intron loss rate across sites, and reconstructed intron loss/gain evolution on each set using four of the five methods (Dollo, RG, Csűrös, EREM). We considered a four-taxa case in which taxa A and B are sisters, and taxa C and D are sisters (Additional data file 2), and in which there were 1,000 introns in CORs in the common ancestor and allowed loss rates to vary between intron positions (Figure 6). In these simulated data sets no parallel gain was allowed to occur.

There are four clear observations, each of which held over all sets of parameters. First, all methods underestimated ancestral intron density. Second, for each data set RG was closest to the real value, followed by EREM, then by Csűrös, then by Dollo parsimony. Third, the Csűrös and EREM methods consistently estimated significant numbers of parallel insertions even though none were included in the simulations - that is, both methods overestimated parallel insertions. Fourth, these trends typically increased with overall branch length. An exception to this was the lack of clear dependency of EREM on branch length.

Together, these observations suggest the following explanation for the discrepancy between previous and current estimates. In the previous data sets [19], the fungi were represented by only *S. pombe *and *S. cerevisiae*, both of which have lost the vast majority of their ancestral introns (that is, the fungal branch was very long). Under such long branch conditions, the RG method somewhat underestimated ancestral intron density, while the other methods considerably underestimated intron density and overestimated parallel insertion. In the new data set, the inclusion of fungal species that retain many more of their ancestral introns shortened the fungal branch, leading to a convergence of the four methods on better estimates (and less or no overestimation of parallel gain by NYK and Csűrös).

Indeed, the difference between NYK's estimation of the incidence of parallel gain between the present and previous data sets is striking. According to the NYK method of calculating parallel intron insertions, our data set showed very little evidence for parallel intron gain. Their method estimated 93.08 total parallel gains; thus, only 2.2 % of 4,228 shared introns were due to parallel gain. This is much less than the previous estimate that 18.5% of shared positions in the Rogozin data set were due to parallel gains. This is despite the fact that the overall number of estimated intron gains, as well as the overall number of estimated gains per kb, was higher in our data set than in the Rogozin data set. Thus, it seems that parallel gains were previously overestimated, and given the near identity of results from Csűrös method to NYK's, the same is very likely true of Csűrös' method.

This decrease in the estimated incidence of parallel gain is all the more striking given the increased number of taxa across data sets, which presumably brings with it an increased number of real gains and real parallel gains, although the implications are not entirely clear given that the species present in the current data set are not a superset of the species in the previous set. Our simulations suggest here that there will be countervailing effects of greater taxonomic sampling, with a decrease in the overestimation of parallel gains due to long-branch effects coinciding with an increase in the overall number of true parallel gains. The decrease in estimated incidence of parallel gain seen here implies that currently the former effect dominates; however, with better and better sampling the latter effect may come to dominate in future data sets. More thorough simulation studies will be necessary to more completely understand this issue.

What of other ancestral nodes of key biological interest for which the different methods gave very different estimates? The three methods' previous estimates based on the Rogozin data set also differed significantly for the fungi-animal-plant ancestor and the bilateran ancestor. In the previous data set, both ancestors were flanked by at least one very long branch, suggesting that all methods might have underestimated intron densities. The finding of intron-rich protostomes and apicomplexans would make resolution of this issue possible in the near future. This argument suggests that intron density was very high even in very early eukaryote ancestors.

## Conclusion

These results resolve a debate over the intron density of the fungal-animal ancestor. All proposed methodologies now agree that this ancestor was very intron rich, and that all modern fungi have experienced more intron loss than gain since divergence. These results underscore that intron evolution in eukaryotic evolution often defies common assumptions of organismal and gene structure complexity and requires new models of intron loss and gain evolution.

## Materials and methods

### Genome data and annotation

Annotated genomes of many of the fungi analyzed were obtained from GenBank or directly from sequencing centers and are listed in Additional data file 3. For unannotated genomes, gene predictions were generated using a combination of *ab initio *and evidence based gene predictions and combined into a single composite gene call with the tool GLEAN [42]. The *ab initio *gene prediction programs SNAP [43], AUGUSTUS [44], and Genezilla [45] were first trained on a set of genes for each genome based on alignments of conserved fungal proteins to the genome using Genewise [46] and Exonerate [47]. At the start of this study, high quality annotations of *Aspergillus fumigatus*, *Aspergillus terreus*, *Coprinus cinereus*, *Podospora anserina *and *Rhizopus oryzae *were not available so automated annotations were generated so that these species could be included. We generated a new annotation of the v1 *P. chrysosporium *genome [13] as we found the previously published gene structures were not of sufficient quality based on multiple sequence alignments of the proteins with other fungal proteins. Prediction parameters derived from the closest annotated species were used with at least one round of retraining as previously described [43]. Frozen versions of genome sequences, annotations in GFF format, Genome Browser [48] and Web BLAST [49] interface to the genomes, predicted coding sequences and proteins are available for download from the authors' site [50].

### Ortholog processing and intron to alignment mapping

The predicted proteins from the 21 fungal genome annotations (Additional data file 3), were combined with the *A. thaliana *annotations (Feb 2005) [4] available from GenBank and the *Fugu rubripes *(Ensembl 30.2e, assembly 2) [51], *Mus musculus *(Ensembl 30.33f) [52], and *H. sapiens *annotations (Ensembl 30.35c) [1]. The longest transcript was used for genes with multiple isoforms. The protein set was masked for low complexity sequences with pseg [53] searched in an all-against-all fashion using FASTP [54] with an expectation value cutoff of 1 × 10^-5^. The output was processed with a custom Perl script to generate, for each pair of species, pairwise orthologs via best-mutual-FASTP hits. The pairwise orthologs were combined via single-linkage clustering for all sets of species into multi-way orthologs only if they formed clusters that contained exactly one protein member from each species.

The protein sequences for these orthologs were then aligned using the multiple sequence alignment program MUSCLE [55]. The protein alignments were used as a guide to align the genes' coding sequences and intron positions were mapped into both the protein and coding sequence alignments using Perl language modules from BioPerl [56]. The 5' and 3' ends of most genes were not alignable and many introns that occurred in these regions, in particular most of the hemiascomycete introns that tend to be within the first few codons of a gene, could not be considered in this study. Alignments of the orthologs are provided as Additional data file 8.

The alignments were evaluated for these intron positions in order to build a matrix of all intron positions. Similar to methodology in previous work [18], each observed intron column in the alignment was classified as to which species shared that intron position. Additionally, an intron position was classified as 'gapped' and removed from the final data matrix if it was within six nucleotides of a column with gaps following methodology from previous studies [19]. The aligned data with intron positions inserted are available in Additional data file 7.

### Phylogenetic analyses

A random sampling of 30 of the protein alignments were used to generate a species tree by concatenating the aligned sequences and removing all gap columns from the alignment. The tree was computed and bootstrapped with MrBayes [57]. The fungal species tree topology was constrained so that *Stagonospora nodorum *is basal to the euascomycetes for consistency with more exhaustive phylogenetic methods using larger sampling of taxa [58]. Other than this constraint, the phylogenetic reconstruction was consistent with other studies that used a larger sampling of orthologous gene sequences [59].

Dollo parsimony was computed with dollop from the PHYLIP package [60] using default parameters. We generated 1,000 bootstrap replicates with seqboot and Dollo parsimony was recomputed on the replicates. The strict consensus tree was computed from these trees with consense in PHYLIP.

### Ancestral intron density reconstruction

The resulting matrix of classified intron positions was evaluated using the RG method computed along the species tree to compute intron densities, numbers of intron gains and losses, and the fraction of introns present at different internal nodes in the tree. The NYK method was also used to construct intron loss and gain rates and densities in ancestral nodes after modification of the authors' C code. The modified RG Perl code and the NYK C code is available in Additional data file 6. The Csűrös method, implemented in intronRates.jar program [16,61], was applied to the data set and allowed to find the optimal number of all-zero unobserved sites. EREM and Dollo parsimony values were computed with the EREM program [35,62]. The EREM values were computed under a homogenous model. The values reported in Figure 5 represent the maximum likelihood estimate from the EREM program of the numbers of predicted introns and gains and losses. Reconstructed values from all five methods are reported in the tables found in Additional data files 4 and 5. No overall comparisons between methods was made for the 'Crown' node or branches leading from this node as not all methods estimate ancestral density or rates without an outgroup.

### Simulations

We simulated a four-taxa case in which taxa A and B are sisters, and taxa C and D are sisters, and in which there were 1,000 introns in CORs in the common ancestor (Additional data file 2). Different introns were assigned different loss rates as given by a standard gamma distribution, with varying gamma-values. The internal branch was set to length zero (neither intron loss nor gain along the internal branch). External branch lengths were set to be of equal length, with a length chosen for each gamma value such that, on average, a given fraction (70% or 30%) of all introns present at the ancestral node were retained in each descendent taxon. We generated data sets for gamma values from 2.0 (most variation in intron loss rate) to 10.0 (least) in increments of 0.5. No insertion, parallel or otherwise, was assumed. For each set of parameters we generated expected numbers of introns with each phylogenetic distribution, and used these values, rounded to the nearest integer, as inputs for all three methods.

## Abbreviations

COR, conserved orthologous region; EREM, evolutionary reconstruction by expectation-maximization; NYK, Nguyen, Yoshihama, and Kenmochi method of intron reconstruction; RG, Roy-Gilbert method of intron reconstruction.

## Authors' contributions

JES conceived of the project, collected the genome data and annotations, annotated genomes, wrote and ran necessary software, performed analyses, created the figures and tables, and wrote the paper. FSD contributed to the content and writing of the paper, provided access to computational resources, and supervised JES. SWR provided intron reconstruction methods, conceived and performed the simulations, analyzed the data, created figures and tables, and wrote the paper.

## Additional data files

The following additional data are available with the online version of this paper. Additional data file 1 is a comparison of two cladograms for the 25 species. Additional data file 2 shows the phylogenetic tree used in the simulation data analysis. Additional data file 3 provides the genomes and annotations used for this analysis with the source and version of the annotation indicated, with references for previously published annotations. Additional data file 4 lists the intron reconstruction values for each node on the tree using the five methods from Nguyen *et al*. [17], Csűrös [16], Roy and Gilbert [18,20], EREM from Carmel *et al*. [35], and Dollo parsimony as computed by EREM. Additional data file 5 lists the rates and numbers of gains and loss for each branch on the tree using the four methods from Nguyen *et al*. [17], Csűrös [16], Roy and Gilbert [18,20], EREM from Carmel *et al*. [35], and Dollo parsimony as computed by EREM. Additional data file 6 provides ummary statistics for intron length, intron frequency, total length per genome, total intron count, total length of coding sequence, and genome size. Additional data file 7 is a zip file containing data for the matrix of intron positions used for this analysis and phylogenetic tree representing the species; the file also contains the customized software for running NYK and the RG intron calculations. Additional data file 8 is a zip file containing multi-FASTA alignments of orthologous genes with introns inserted into protein alignments.

## Supplementary Material

Additional data file 1 The left tree was built with a MrBayes using 30 orthologous proteins with the position of *S. nodorum *constrained based on previously published phylogenies. The tree on the right is the strict consensus tree of 116 MP trees built using Dollo parsimony and the matrix of presence or absence of intron positions. Nodes that are not present in all 116 trees are collapsed. Species groups are colored so that Euascomycota are in dark green, Hemiascomycota in red, archiascomycete *S. pombe *in yellow, Basidiomycota excluding *U. maydis *in blue, *U. maydis *in purple, zygomycete *R. oryzae *in orange, vertebrates in pink, and green plant *A. thaliana *in light green. Click here for file

Additional data file 2 The phylogenetic tree used in the simulation data analysis. Click here for file

Additional data file 3 Genomes and annotations used for this analysis with the source and version of the annotation indicated, with references for previously published annotations. Click here for file

Additional data file 4 Not all methods reconstruct a value for the Crown ancestor as this requires an outgroup and additional assumptions. Click here for file

Additional data file 5 Not all methods estimate gain and loss from Crown ancestor as this requires an outgroup and additional assumptions. Click here for file

Additional data file 6 Summary statistics for intron length, intron frequency, total length per genome, total intron count, total length of coding sequence, and genome size. Click here for file

Additional data file 7 The file also contains the customized software for running NYK and the RG intron calculations. Click here for file

Additional data file 8 Introns are represented by numbers in the alignment indicating the phase of the intron (0,1,2) as defined by the position in the codon the intron falls within. Coding sequence alignments and unaligned sequences are available from the authors. Click here for file
