# Supplementary material for: Comparative genomic analysis of fungal genomes reveals intron-rich ancestors
Source: Genome Biol. 2007 Oct 19;8(10):R223. doi: 10.1186/gb-2007-8-10-r223 (PMC2246297; doi:10.1186/gb-2007-8-10-r223)
Supplement: Additional data file 3 — Genomes and annotations used for this analysis with the source and version of the annotation indicated, with references for previously published annotations. [file gb-2007-8-10-r223-S3.doc]

Table S1: Genomes and annotations used in the study

| **Species** | **Sequencing Center or Source** | **Citation(s)** | **Assembly version** | **Annotation** |
| --- | --- | --- | --- | --- |
| *Ashbya gossypii* | Biozentrum | [1] | finished | GenBank |
| *Aspergillus fumigatus* | TIGR | [2] | Sep 2004 | this study |
| *Aspergillus nidulans* | Broad Institute/FGI | [3] | v1; Mar 2003 | Broad Institute (v1) |
| *Aspergillus terreus* | Broad Institute/FGI | unpublished | v1; Sep 2005 | this study |
| *Chaetomium globsum* | Broad Institute/FGI | unpublished | Jan 2004 | Broad Institute |
| *Coprinus cinereus* | Broad Institute/FGI | unpublished | v1; Jun 2003 | this study |
| *Cryptococcus neoformans* | TIGR | [4] | Jan 2005 | GenBank |
| *Candida glabrata* | Génolevures | [5] | Jul 2004 | GenBank |
| *Debaryomyces hansenii* | Génolevures | [5] | Jul 2004 | GenBank |
| *Fusarium graminearum* | Broad Institute/FGI | unpublished | v1 | Broad Institute |
| *Kluyveromyces lactis* | Génolevures | [5] | Jul 2004 | GenBank |
| *Magnaporthe grisea* | Broad Institute/FGI | [6] | v2; Oct 2003 | Broad Institute (v4) |
| *Neurospora crassa* | Broad Institute/FGI | [7] | v3 | Broad Institute (v3) |
| *Phanerochaete chrysosporium* | JGI | [8] | v1 (Feb 2002) | this study |
| *Podospora anserina* | CNRS/Génoscope | unpublished | Jan 2004 | this study |
| *Rhizopus oryzae* | Broad Institute/FGI | unpublished | v1 | this study |
| *Saccharomyces cerevisiae* | SGD | [9] | Jan 2005 | SGD |
| *Schizosaccharomyces pombe* | Sanger Centre | [10] | 2004 | EMBL |
| *Stagonospora nodrum* | Broad Institute/FGI | unpublished | v1; Feb 2005 | Broad Institute |
| *Ustilago maydis* | Broad Institute/FGI | [11] | v1; Nov 2003 | Broad Institute/MIPS (v1) |
| *Yarrowia lipolytica* | Génolevures | [5] | Jul 2004 | GenBank |
| *Arabidopsis thaliana* | TIGR | [12] | Feb 2005 | TIGR |
| *Fugu rubripes* | Ensembl | [13] | Assembly 2 | Ensembl 30.2e |
| *Homo sapiens* | Ensembl | [14] | NCBI 35 | Ensembl 30.35c |
| *Mus musculus* | Ensembl | [15] | NCBI 33 | Ensembl 30.33f |

Abbreviations are as follows.

Broad Institute=Broad Institute of MIT and Harvard, Cambridge, MA USA.

CNRS=Centre National de la Recherche Scientifique, Paris, France.

Ensembl=Ensembl project of European Bioinformatics Institute and Sanger Centre, UK.

FGI=Fungal Genome Initiative.

Génolevures=large-scale comparative genomics project between *Saccharomyces cerevisiae* and 14 other yeast species <http://cbi.labri.fr/Genolevures/about.php>.

Génoscope=Génoscope Paris, France.

JGI=Joint Genome Institute of US Department of Energy, Walnut Creek, CA USA.

MIPS=Munich Information Center for Protein Sequences, Munich, Germany.

Sanger Centre=Welcome Trust Sanger Centre, Hinxton, UK.

TIGR=The Institute for Genome Research, Rockville, MD USA.

The annotations generated for this study are available from the website [http://fungal.genome.duke.edu](http://fungal.genome.duke.edu/) in GFF format.

1. Dietrich FS, Voegeli S, Brachat S, Lerch A, Gates K, Steiner S, Mohr C, Pohlmann R, Luedi P, Choi S *et al*: **The *Ashbya gossypii* genome as a tool for mapping the ancient Saccharomyces cerevisiae genome**. *Science* 2004, **304**(5668):304-307.

2. Nierman WC, Pain A, Anderson MJ, Wortman JR, Kim HS, Arroyo J, Berriman M, Abe K, Archer DB, Bermejo C *et al*: **Genomic sequence of the pathogenic and allergenic filamentous fungus *Aspergillus fumigatus***. *Nature* 2005, **438**(7071):1151-1156.

3. Galagan JE, Calvo SE, Cuomo C, Ma LJ, Wortman JR, Batzoglou S, Lee SI, Basturkmen M, Spevak CC, Clutterbuck J *et al*: **Sequencing of *Aspergillus nidulans* and comparative analysis with *A. fumigatus* and *A. oryzae***. *Nature* 2005, **438**(7071):1105-1115.

4. Loftus BJ, Fung E, Roncaglia P, Rowley D, Amedeo P, Bruno D, Vamathevan J, Miranda M, Anderson IJ, Fraser JA *et al*: **The genome of the basidiomycetous yeast and human pathogen *Cryptococcus neoformans***. *Science* 2005, **307**(5713):1321-1324.

5. Dujon B, Sherman D, Fischer G, Durrens P, Casaregola S, Lafontaine I, De Montigny J, Marck C, Neuveglise C, Talla E *et al*: **Genome evolution in yeasts**. *Nature* 2004, **430**(6995):35-44.

6. Dean RA, Talbot NJ, Ebbole DJ, Farman ML, Mitchell TK, Orbach MJ, Thon M, Kulkarni R, Xu JR, Pan H *et al*: **The genome sequence of the rice blast fungus *Magnaporthe grisea***. *Nature* 2005, **434**(7036):980-986.

7. Galagan JE, Calvo SE, Borkovich KA, Selker EU, Read ND, Jaffe D, FitzHugh W, Ma LJ, Smirnov S, Purcell S *et al*: **The genome sequence of the filamentous fungus *Neurospora crassa***. *Nature* 2003, **422**(6934):859-868.

8. Martinez D, Larrondo LF, Putnam N, Gelpke MD, Huang K, Chapman J, Helfenbein KG, Ramaiya P, Detter JC, Larimer F *et al*: **Genome sequence of the lignocellulose degrading fungus *Phanerochaete chrysosporium* strain RP78**. *Nat Biotechnol* 2004, **22**(6):695-700.

9. Hirschman JE, Balakrishnan R, Christie KR, Costanzo MC, Dwight SS, Engel SR, Fisk DG, Hong EL, Livstone MS, Nash R *et al*: **Genome Snapshot: a new resource at the Saccharomyces Genome Database (SGD) presenting an overview of the *Saccharomyces cerevisiae* genome**. *Nucleic Acids Res* 2006, **34**(Database issue):D442-445.

10. Wood V, Gwilliam R, Rajandream MA, Lyne M, Lyne R, Stewart A, Sgouros J, Peat N, Hayles J, Baker S *et al*: **The genome sequence of *Schizosaccharomyces pombe***. *Nature* 2002, **415**(6874):871-880.

11. Kamper J, Kahmann R, Bolker M, Ma LJ, Brefort T, Saville BJ, Banuett F, Kronstad JW, Gold SE, Muller O *et al*: **Insights from the genome of the biotrophic fungal plant pathogen Ustilago maydis**. *Nature* 2006, **444**(7115):97-101.

12. Haas BJ, Wortman JR, Ronning CM, Hannick LI, Smith RK, Jr., Maiti R, Chan AP, Yu C, Farzad M, Wu D *et al*: **Complete reannotation of the *Arabidopsis* genome: methods, tools, protocols and the final release**. *BMC Biol* 2005, **3**(1):7.

13. Aparicio S, Chapman J, Stupka E, Putnam N, Chia JM, Dehal P, Christoffels A, Rash S, Hoon S, Smit A *et al*: **Whole-genome shotgun assembly and analysis of the genome of *Fugu rubripes***. *Science* 2002, **297**(5585):1301-1310.

14. Collins FS, Lander ES, Rogers J, Waterston RH, International Human Genome Sequencing Consortium: **Finishing the euchromatic sequence of the human genome**. *Nature* 2004, **431**(7011):931-945.

15. Waterston RH, Lindblad-Toh K, Birney E, Rogers J, Abril JF, Agarwal P, Agarwala R, Ainscough R, Alexandersson M, An P *et al*: **Initial sequencing and comparative analysis of the mouse genome**. *Nature* 2002, **420**(6915):520-562.
